# Pharmacokinetic Properties of the Novel Synthetic Cannabinoid 5F-APINAC and Its Influence on Metabolites Associated with Neurotransmission in Rabbit Plasma

**DOI:** 10.3390/ph14070668

**Published:** 2021-07-13

**Authors:** Ksenia M. Shestakova, Natalia V. Mesonzhnik, Pavel A. Markin, Natalia E. Moskaleva, Andrey A. Nedorubov, Alex Brito, Elizaveta G. Appolonova, Roman M. Kuznetsov, Natalia L. Bochkareva, Alexey Kukharenko, Alexey V. Lyundup, Franco Tagliaro, Svetlana A. Appolonova

**Affiliations:** 1 Laboratory of Pharmacokinetics and Metabolomic Analysis, Institute of Translational Medicine and Biotechnology, I.M. Sechenov First Moscow State Medical University, 119991 Moscow, Russia; ksenia.shestakova@labworks.ru (K.M.S.); natalia.mesonzhnik@labworks.ru (N.V.M.); markinpavel96@gmail.com (P.A.M.); nemoskaleva@gmail.com (N.E.M.); abrito@labworks.ru (A.B.); roman.kuznetsov@labworks.ru (R.M.K.); natalia.bochkareva@labworks.ru (N.L.B.); alexey.kukharenko@labworks.ru (A.K.); franco.tagliaro@univr.it (F.T.); 2 PhD Program in Nanosciences and Advanced Technologies, University of Verona, 37129 Verona, Italy; 3 World-Class Research Center “Digital Biodesign and Personalized Healthcare”, I.M. Sechenov First Moscow State Medical University, 119991 Moscow, Russia; 4 Russian Center of Forensic-Medical Expertise of the Ministry of Health, 125284 Moscow, Russia; 5 Center for Preclinical Research, Institute of Translational Medicine and Biotechnology, I.M. Sechenov First Moscow State Medical University, 119991 Moscow, Russia; nedorubov.ras@gmail.com; 6 A. Butlerov Institute of Chemistry, Kazan Federal University, Kazan, 420008 Republic of Tatarstan, Russia; elisabetappolonova@yandex.ru; 7 Research and Educational Resource Center for Cellular Technologies of Peoples’ Friendship University of Russia, 117198 Moscow, Russia; lyundup@gmail.com; 8 Unit of Forensic Medicine, Department of Diagnostics and Public Health, University of Verona, 37129 Verona, Italy

**Keywords:** synthetic cannabinoids, 5F-APINAC, UPLC-MS/MS, pharmacokinetics, neurotransmitters, metabolites, metabolomics

## Abstract

The strong psychoactive effects of synthetic cannabinoids raise the need for the deeper studying of their neurometabolic effects. The pharmacokinetic properties of 5F-APINAC and its influence on metabolomics profiles associated with neurotransmission were investigated in rabbit plasma. Twelve rabbits divided into three groups received 1-mL 5F-APINAC at 0.1, 1 and 2 mg/kg. The intervention groups were compared with the controls. Sampling was performed at nine time points (0–24 h). Ultra-high-performance liquid chromatography–tandem mass spectrometry was used. The pharmacokinetics were dose-dependent (higher curve at a higher dose) with a rapid biotransformation, followed by gradual elimination within 24 h. The tryptophan concentrations abruptly decreased (*p* < 0.05) in all tested groups, returning to the basal levels after 6 h. 5-hydroxylindole acetic acid increased (*p* < 0.05) in the controls, but this trend was absent in the treated groups. The aspartic acid concentrations were elevated (*p* < 0.001) in the treated groups. L-kynurenine was elevated (*p* < 0.01) in the intervention groups receiving 1 mg/kg to 2 mg/kg. Dose-dependent elevations (*p* < 0.01) were found for kynurenic acid, xanthurenic acid and quinolinic acid (*p* < 0.01), whereas the anthranilic acid trends were decreased (*p* < 0.01). The indole-3-propionic acid and indole-3-carboxaldehyde trends were elevated (*p* < 0.05), whereas the indole-3-lactic acid trajectories were decreased (*p* < 0.01) in the intervention groups. 5F-APINAC administration had a rapid biotransformation and gradual elimination. The metabolites related to the kynurenine and serotonergic system/serotonin pathways, aspartic acid innervation system and microbial tryptophan catabolism were altered.

## 1. Introduction

The illegal use of synthetic cannabinoids (SCs) has been growing exponentially [1,2,3]. These drugs represent one of the largest groups of new psychoactive substances (NPS), gaining significant importance in clinical and forensic toxicology. SCs are characterized by a strong affinity to cannabinoid receptors (CB1 and CB2) and by a highly lipophilic nature. Most SCs easily cross the blood–brain barrier, having access to the CB and receptors displaying a binding to them up to 100 times stronger than their natural analog, ∆9-tetrahydrocannabiol (THC). The affinity of SCs to CB receptors results in strong psychoactive “cannabimimetic” effects, even at low doses of consumption. Moreover, SCs may cause severe alterations, including neurological, cardiovascular and gastrointestinal disorders [4]. Within neurons, CB1 receptors are often localized at axon terminals, and their activation leads to the inhibition of neurotransmitter releases [5]. These receptors may interact with ion channels, exploiting their inhibition or activation [6].

The novel SC 5F-APINAC represents a recently emerged NPS belonging to an adamantylindazole structural class [7]. The in vitro and in vivo metabolism of 5F-APINAC in rats has been described postulating the rapid hydrolysis and hydroxylation of the parent drug with a consequent formation of its main metabolites—5F-pentylidazol carboxylic acid and hydroxyl derivatives [7]. Notably, due to the rapid metabolism of 5F-APINAC, the parent drug has not been detected so far in urine. In a zebrafish model, the short- and long-term exposures of 5F-APINAC induced metabolomic alterations associated with neurotransmitter systems and embryotoxicity confirmed by teratogenicity [8]. However, to our knowledge, the pharmacokinetics properties of 5F-APINAC, as well as the influence of this drug on mammal neurotransmitter metabolisms, have not been characterized yet. Considering the potential risks associated with the 5F-APINAC intake, a pharmacokinetic and metabolomics assessment of this drug is needed for a deeper understanding of the 5F-APINAC toxicity, as well as for the better monitoring of its consumption in clinical and forensic contexts [9,10,11]. Considering the present lack of knowledge in the relation between the blood concentrations of 5F-APINAC and its toxic effects, we hypothesized that exposure to 5F-APINAC could produce dose-dependent curves and drug-specific quantifiable changes in the metabolites associated with neurotransmission (i.e., those formed by the tryptophan biodegradation pathway). Thus, the present study was undertaken with the aim of characterizing the pharmacokinetic properties of 5F-APINAC and its influence on the targeted metabolomics profile associated with neurotransmission in rabbit plasma.

## 2. Results

### 2.1. Rabbit Plasma Pharmacokinetic Properties of 5F-APINAC

**Figure 1:**
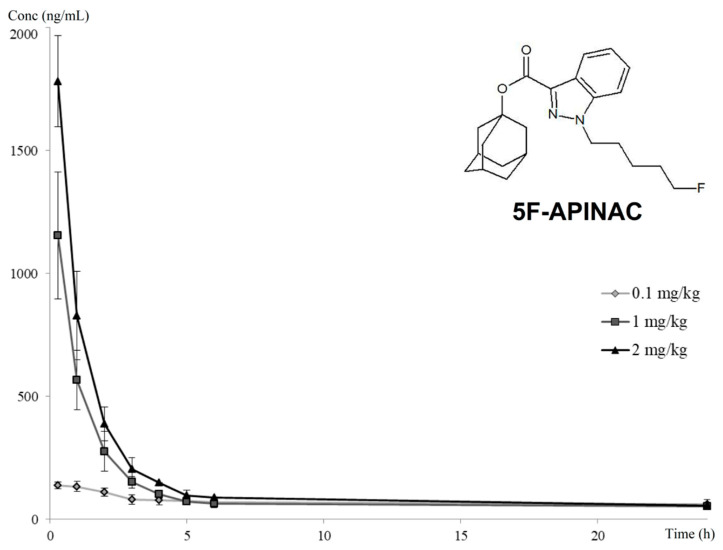
Pharmacokinetics profile of 5F-APINAC after the intravenous administration of 0.1, 1 and 2-mg/kg dosages in rabbit plasma. Blood samples were taken at 20 min and at 1, 2, 3, 4, 5, 6 and 24 h after the drug administration.

The pharmacokinetics profile was characterized by being dose-dependent (higher curve at a higher dose) with rapid biotransformation within the first five hours after drug administration, followed by gradual elimination within 24 h (Figure 1).

To assess the pharmacokinetic properties of 5F-APINAC at different dosages, we calculated: the half-life time (t1/2), area under the pharmacokinetic curve (AUC 0–24), the mean residual time in the bloodstream (MRT 0–24) and the clearance. The received pharmacokinetic parameters for all the dosages are presented in Table 1.

### 2.2. Targeted Metabolomics Profile Associated with Neurotransmission

Thirty-six metabolites were quantified. Table 2 summarizes the main findings for the targeted metabolites associated with neurotransmission. For the visualization of the endogenous metabolites after 5F-APINAC administration, a heat map and clustering analysis of the extracted metabolites were made to present the discrimination between the interventions and vehicle control groups (Supplementary Figure S1A–D).

#### 2.2.1. Serotonergic System-Serotonin Pathway

**Figure 2:**
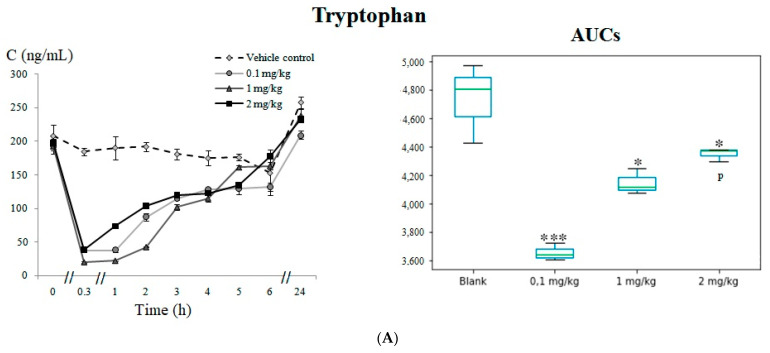

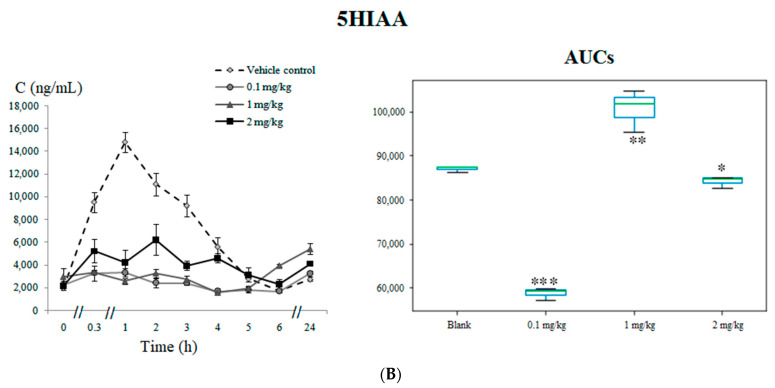
Metabolites related with the serotonergic system–serotonin pathway, including: (**A**) time-concentration profile and boxplots of the AUC for tryptophan and (**B**) time-concentration profile and boxplots of the AUC for 5-Hydroxylindole acetic acid. Statistically significant differences in the AUCs between the vehicle control versus the test groups are marked with asterisks as * *p* < 0.05, ** *p* < 0.01 and *** *p* < 0.001.

The concentration levels of tryptophan abruptly decreased (*p* < 0.05) immediately after 5F-APINAC administration in all tested groups of the animals and returned to the basal level after six hours of drug administration (Figure 2A). The metabolite 5-hydroxylindole acetic acid (5HIAA) was increased (*p* < 0.05) at the first hour in the vehicle control group. However, this trend was not observed in any of the treated groups versus the vehicle control (Figure 2B).

#### 2.2.2. Aspartic Acid Innervation System

**Figure 3:**
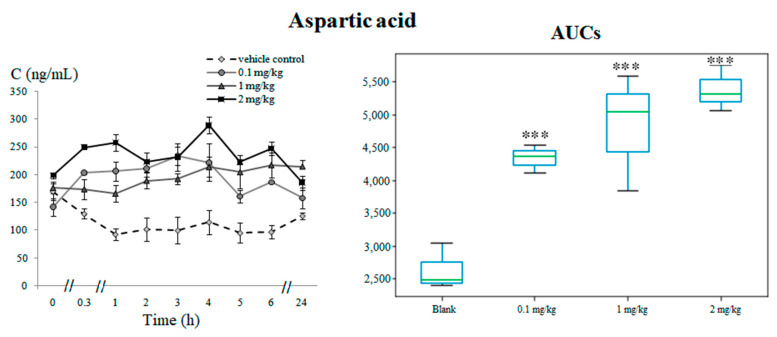
Aspartic acid, a metabolite related with the aspartic acid innervation system. Statistically significant differences in the AUCs between the vehicle control versus the test groups are marked with asterisks as *** *p* < 0.001.

The only metabolite found to be significantly altered in the aspartic acid innervation system was aspartic acid, which was found to be consistently elevated (*p* < 0.001) in all the treated groups of rabbits compared with the vehicle control group (Figure 3).

#### 2.2.3. Kynurenine Pathway

**Figure 4:**
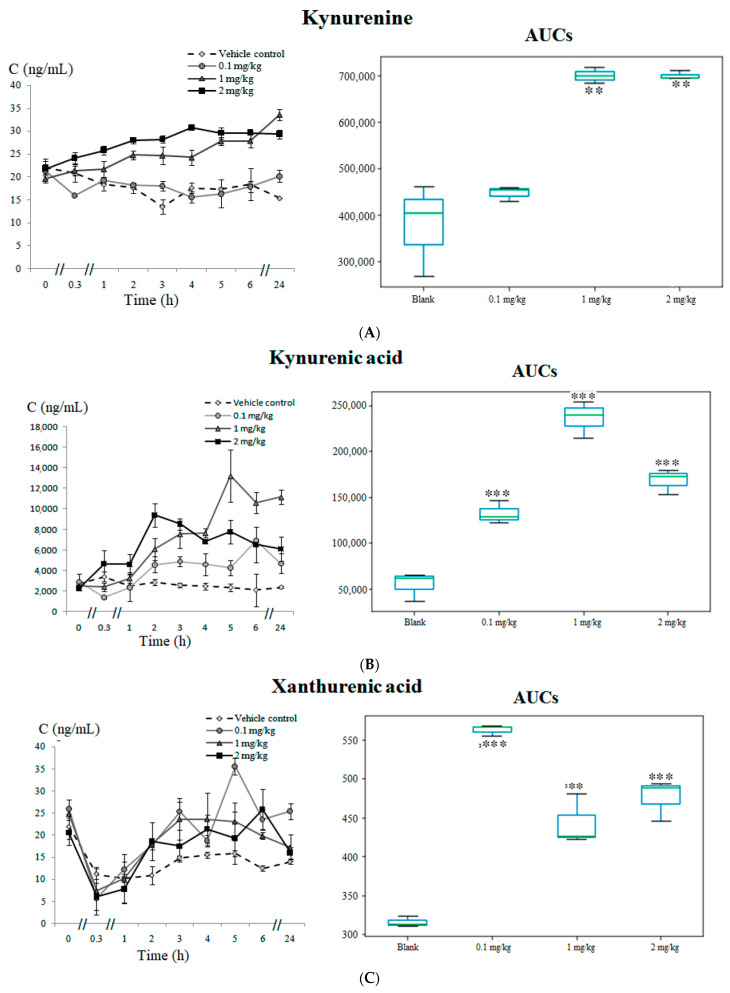

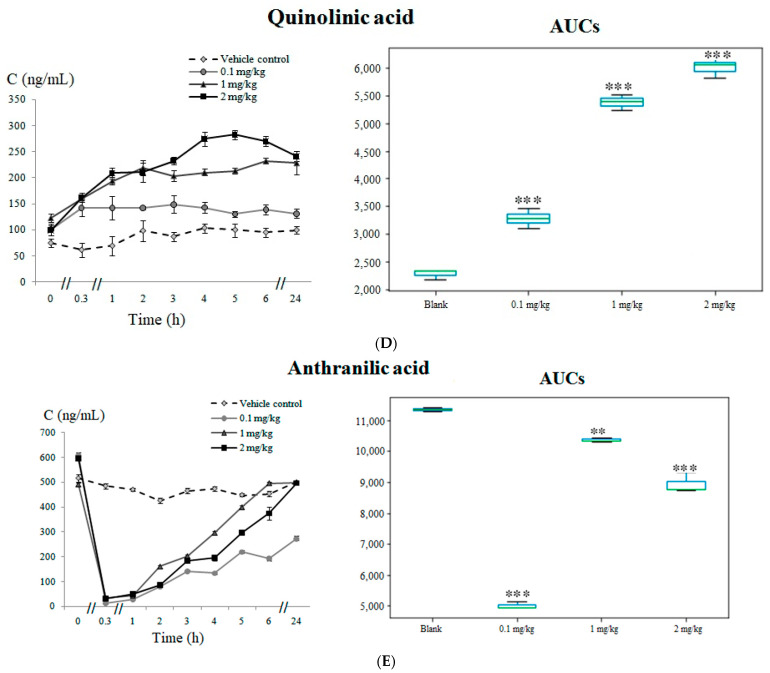
Metabolites related with the kynurenine pathway: (**A**) time-concentration profile and boxplots of the AUC for kynurenine, (**B**) time-concentration profile and boxplots of the AUC for kynurenic acid, (**C**) time-concentration profile and boxplots of the AUC for xanthurenic acid, (**D**) time-concentration profile and boxplots of the AUC for quinolinic acid and (**E**) time-concentration profile and boxplots of the AUC for anthranilic acid. Statistically significant differences in the AUCs between the vehicle control versus the test groups are marked with asterisks as ** *p* < 0.01 and *** *p* < 0.001.

The kynurenine pathway was the neurotransmitter system with the highest number of metabolites significantly altered as a consequence of 5F-APINAC administration. L-kynurenine, one of the main biodegradation products of tryptophan, was elevated (*p* < 0.01) in the intervention groups of the rabbits that received 1 mg/kg and 2 mg/kg of 5F-APINAC (Figure 4A). The tryptophan conversion intermediates kynurenic acid (Figure 4B), xanthurenic acid (Figure 4C) and quinolinic acid (Figure 4D) were also increased (*p* < 0.01) in a dose-dependent manner in the intervention groups compared to the vehicle control, whereas the anthranilic acid trends were decreased (*p* < 0.01) in the intervention groups versus the control (Figure 4E).

#### 2.2.4. Microbial Tryptophan Catabolism

**Figure 5:**
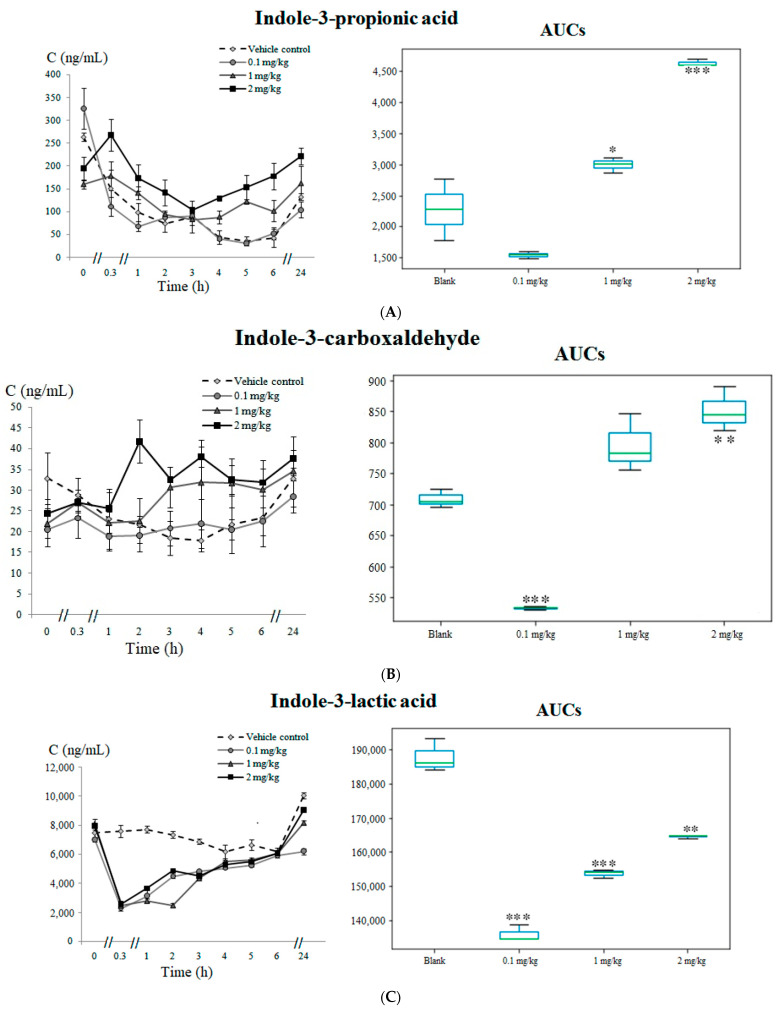
Metabolites related with microbial tryptophan catabolism: (**A**) time-concentration profile and boxplots of the AUC for indole-3-propionic acid, (**B**) time-concentration profile and boxplots of the AUC for indole-3-carboxaldehyde and (**C**) time-concentration profile and boxplots of the AUC for indole-3-lactic acid. Statistically significant differences in the AUCs between the vehicle control and test groups are marked with * *p* < 0.05, ** *p* < 0.01 and *** *p* < 0.001.

The indole-3-propionic acid (Figure 5A) and indole-3-carboxaldehyde (Figure 5B) trends were found to be elevated (*p* < 0.05) in the intervention rabbits, whereas the indole-3-lactic acid (Figure 5C) trajectories were decreased (*p* < 0.01) in all the intervention groups compared to the control.

None of the metabolites related to the gamma-aminobutyric acid/glutamic acid innervation system or the dopaminergic/adrenergic system, and the cholinergic systems presented significant dose-dependent trends across time.

## 3. Discussion

**Figure 6:**
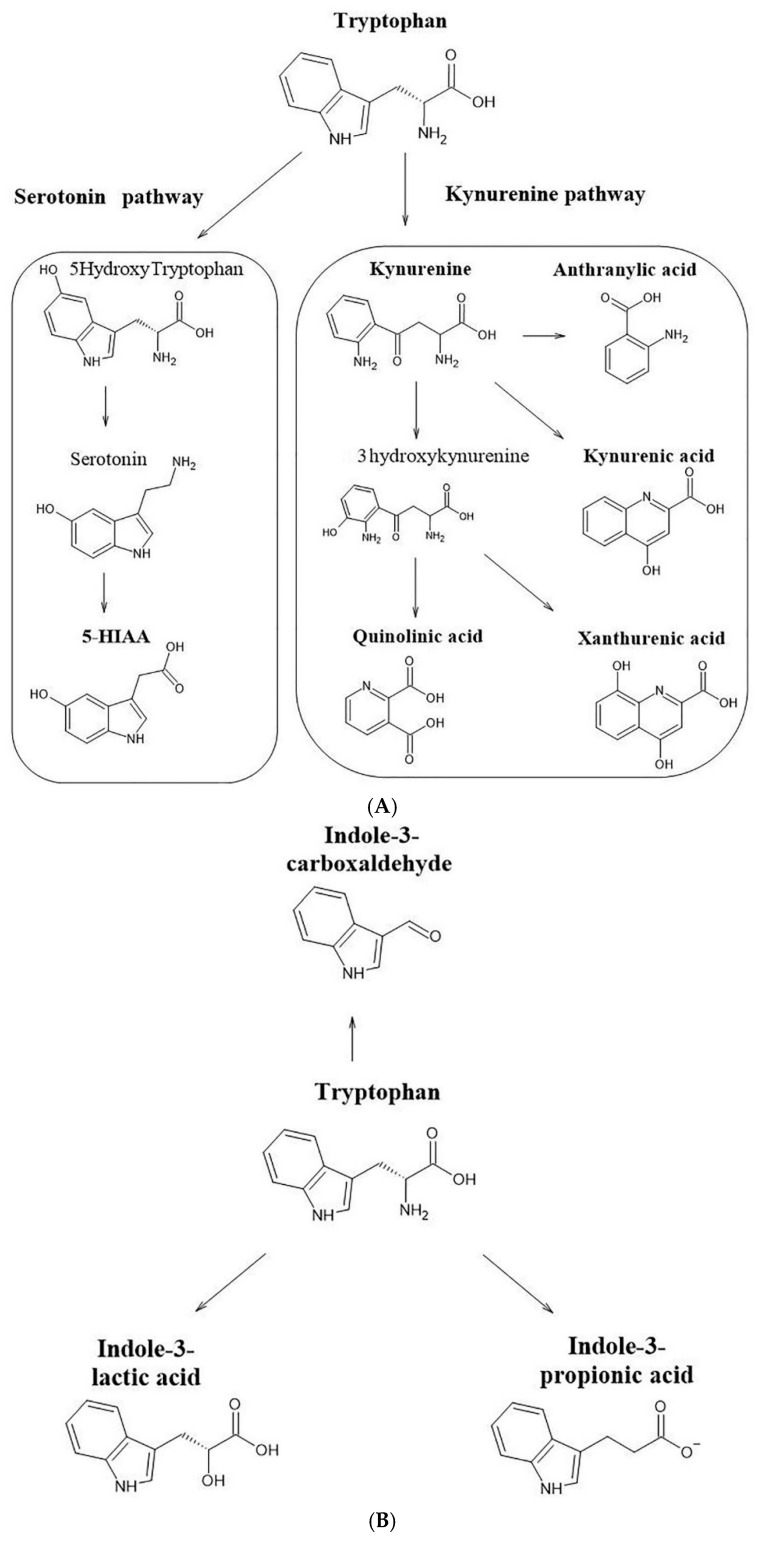

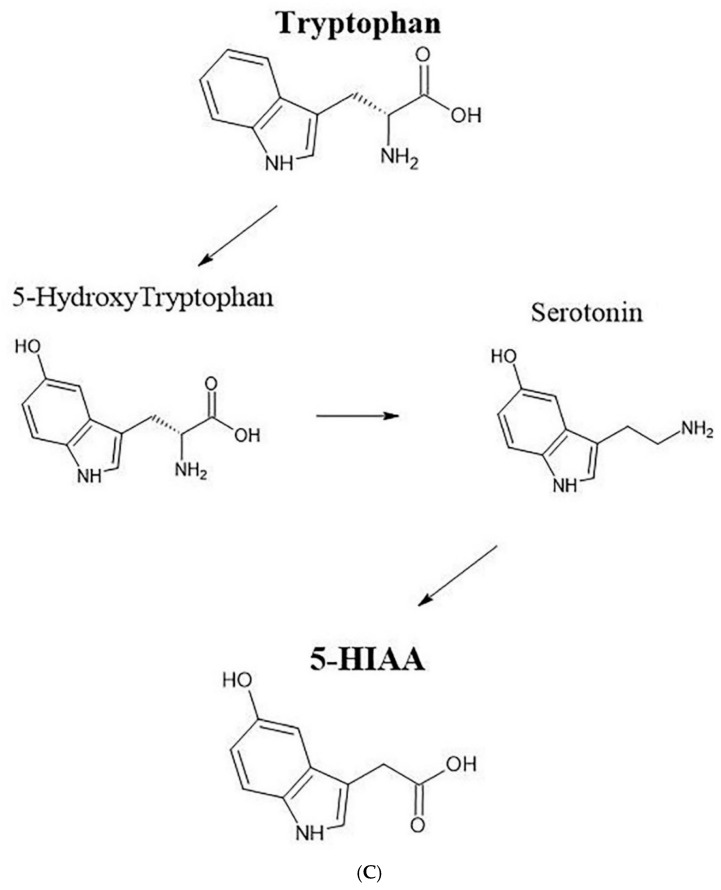
Metabolic pathway diagram summarizing the metabolites altered in response to 5F-APINAC administration: (**A**) the kynurenine metabolic pathway, (**B**) serotonergic system/serotonin pathway, aspartic acid innervation system and (**C**) microbial tryptophan catabolism pathway.

Any new aspect of the knowledge of pharmacokinetics and metabolomics of SCs may serve for a better understanding of the clinical and toxicological findings attributable to drug poisoning and misuse cases [12]. Mass spectrometric approaches are commonly used in the study of endogenous compounds [13]. The current study used a pharmacokinetic approach and targeted metabolomics of metabolites associated with neurotransmission to assess the effects of 5F-APINAC administration in mammals. This study was performed with the aim of investigating the toxicity and possible influence of this molecule on neurotransmission systems. The pharmacokinetic profile of 5F-APINAC was dose-dependent with a rapid biotransformation, followed by a gradual elimination within 24 h. The main metabolic pathways found to be impacted were the kynurenine metabolic pathway, serotonergic system/serotonin pathway, aspartic acid innervation system and microbial tryptophan catabolism (Figure 6A–C).

It should be pointed out that, due to the low doses typical of SCs consumption, as well as their highly extensive metabolism, the pharmacokinetics assessments of SCs are complex. Only a few experiments describing the pharmacokinetic profiles of SCs in animal models have been published. The received concentration–time curve was smooth enough for the accurate assessment of the main pharmacokinetic parameters of the drug. The 5F-APINAC maximum concentrations after the 1-mg/kg and 2-mg/kg doses were higher than those after 0.1 mg/kg. The maximum concentration, as well as the AUC values, rose linearly in proportion to the administered dose, indicating linear kinetics. It should also be noted that the 5F-APINAC concentrations were substantially reduced but still detectable 24 h after the drug administration, but the concentration levels were close to the lowest limits of detection.

Since most of the SCs are full agonists of CB receptors, they provide strong neuroactive and toxic effects on the central nervous system (CNS). On the other hand, in the last decades, it has been found that tryptophan metabolism and the metabolites associated with neurotransmission are strongly associated with numerous CNS disorders. Conversely, several tryptophan derivatives have been found to display high levels of neuronal activity, possibly associated with the administration of SCs.

Tryptophan is an essential amino acid known for its influence on immunological and neurological activity, being a precursor for the synthesis of proteins and nicotinamide adenine dinucleotide (NAD), as well as nicotinic acid and serotonin [14]. Moreover, a number of its metabolites may pass the blood–brain barrier, having neuroactive properties on the brain physiology connected to behavioral changes [15,16]. Tryptophan metabolism consists mainly of two competing major metabolic pathways: the kynurenine pathway that converts approximately 95% of tryptophan and the serotonin pathway that metabolizes 5% of the amino acid. We confirmed that both the serotonergic system–serotonin pathway and, especially, the kynurenine pathway were the most-impacted pathways in our experiment. Alterations in tryptophan metabolism may appear following stress or depression. In the case of the kynurenine pathway, this is related to the control of plasma tryptophan levels through the clearance of excess of circulating tryptophan [17]. Additionally, it has been reported that the kynurenine pathway provides metabolites involved in neurotransmission, such as kynurenine, kynurenic acid and quinolinic acid [14].

Several studies have described the effects of THC on tryptophan conversion and the association with behavioral and neurological changes. It has been hypothesized that THC and cannabidiol (CBD) suppress tryptophan degradation through the activation of indoleamine-2,3-dioxygenase. This would result in the enhanced availability of tryptophan for serotonin biosynthesis, which could produce improvements of mood disturbances.

Based on our findings, it may be postulated that, after 5F-APINAC administration, the changes in tryptophan metabolism are strongly associated with the kynurenine pathway. According to the human proteome map, the expression of several enzymes related to the kynurenine pathway are much higher in the liver than in the brain. The liver plays an essential role in amino acid metabolism, being the major organ that metabolizes absorbed dietary tryptophan. The release of tryptophan metabolites into the systemic circulation has hepatic regulation. It may be postulated that precursors and side intermediates for tryptophan metabolism to other organs, including the brain, are provided by the liver, which hereby represents one of the key regulators of kynurenine metabolite levels in the bloodstream [18]. Our results were based on the identification of the biochemical features detected in the blood. We recently reported that the short- and long-term exposures of 5F-APINAC in a zebrafish model were mainly characterized by alterations in the kynurenine pathway but, also, in other neurotransmitter systems such as the gamma-aminobutyric acid/glutamic acid, dopaminergic/adrenergic and cholinergic neurotransmitter systems not found to be altered in rabbit plasma [8]. This may be explained by the fact that these alterations were measured in the entire organism of the zebrafish, reflecting the systemic biochemical alterations from the organs and not just from the blood, as in the present study.

The tryptophan concentrations immediately decreased at all doses within the first two hours after 5F-APINAC administration. It is generally observed that the circulating levels of tryptophan are mainly dependent on its exogenous supply of the amino acid. However, cytokine-induced tryptophan degradation may constitute an additional important mechanism potentially justifying, through the serotonergic system, the anti-depressive effects of cannabinoids [19]. It should be taken into consideration that, as mentioned above, approximately 95% of tryptophan metabolism passes through the kynurenine pathway. In this case, the first step of the pathway is generally affected by the enzyme tryptophan pyrrolase. Its release is activated in stress conditions, resulting in a decrease of blood tryptophan concentrations. Additionally, it has been found in the active conversion of tryptophan into kynurenine in depressed individuals [20].

L-kynurenine represents a central metabolite of the kynurenine pathway. The experimental data showed a dose-dependent increase of plasma concentrations in rabbits receiving 1-mg/kg and 2-mg/kg 5F-APINAC. However, the group of rabbits receiving the lowest dose (0.1 mg/kg) did not present a significant difference compared to the vehicle control group. Due to the ability of transportation through the blood–brain barrier by the neutral amino acid carriers, L-kynurenine may easily reach the central nervous system and be captured and metabolized by the glial cells [21,22]. Notably, Stone et al. hypothesized that kynurenine does not directly affect the neuronal electrical activity in brains or in isolated neurons and has no direct depressant influence on cardiac and smooth muscle tissues [23]. At the same time, kynurenine has been shown to increase the expression and synthesis of the nerve growth factor that plays an importance role in the tropism of different neurons [24]. Kynurenine may be further metabolized along three neurotoxic branches, with the consequent formation of 3-hydroxy-L-kynurenine, kynurenic acid and anthranilic acid. In general, it has been reported that metabolites in the kynurenine pathway take part in different pathophysiological processes, including neurodegenerative and neurological diseases and even psychiatric disorders such as schizophrenia or depression [16].

Kynurenic acid, found to be elevated in the tested groups at all doses (with a maximum increase in a medium concentration) in our study, is generated by the action of a specific enzyme kynurenine aminotransferase and represents one of the most widely studied neuroactive tryptophan metabolites. It is mainly present in the brain, being an antagonist for the receptors of excitatory amino acids [25]. Elevated levels of kynurenic acid have shown neuroprotective and neuroinhibitory properties associated with its action as a competitive antagonist at the glycine site of NMDA receptors [26]. Kynurenic acid inhibits noncompetitive α7-nicotinic acetylcholine receptors, which are the primary endogenous targets of kynurenic acid, and binds α-bungarotoxin, which blocks the action of acetylcholine at the postsynaptic membrane, inhibiting the ion flow [27,28]. Considering the interaction of kynurenic acid with different receptors, as well as its effect on the extracellular levels of glutamate, dopamine, acetylcholine and γ-aminobutyric acid, L. A. Ramos-Chávez et al. considered this molecule a neuromodulator. It is also worth considering that these neurotransmitters take part in neuronal development and plasticity, thus affecting cognition, behavior and memory processes [29]. The regulation of the effects of these neurotransmitters by kynurenic acid may contribute to the knowledge of the mechanisms of the neurological effects of cannabinoids.

The hydroxylation of kynurenine by kynurenine 3-monooxygenase results in the formation of 3-hydroxykynurenine, which is further metabolized into xanthurenic acid and quinolinic acid. Actually, the levels of xanthurenic acid in the test group of rabbits were elevated at all levels of the administered drug, although without any dependence on the 5F-APINAC doses. Despite the fact that it is structurally similar to kynurenic acid, its biological role remains unclear. Several authors have suggested that xanthurenic acid might play a role in neurotransmission, neuromodulation and synaptic signaling, in relation with an active intake by synaptic vesicles in the brain [30]. In the present study, the levels of quinolinic acid found in rabbits administered with 5F-APINAC were also elevated in a dose-dependent manner. Quinolinic acid is a potent excitotoxin, as well as a strong NMDA receptor agonist [14]. By its interaction with NMDA-type glutamate receptors, quinolinic acid has been reported to cause convulsions and other excitatory effects [31]. On the other hand, quinolinic acid causes axon-sparing lesions in the brain, associated with mitochondrial damages and free radical generation [28]. Simultaneously, other neurotoxicity effects of quinolinic acid are associated with reactive oxygen species formation and destabilization of the cytoskeleton [32,33]. Moreover, quinolinic acid toxicity affects oligodendrocytes, neurons and motor neurons [34,35,36]. Thus, elevated levels of quinolinic acid in our study may be connected to the neurotoxic effects of 5F-APINAC.

We found that the concentration levels of aspartic acid were significantly increased in the treated group of animals. This can be explained by the fact that an increase of the concentration of quinolinic acid may cause the release of glutamate and aspartic acid from the cerebral cortex in vivo, which predisposes excitotoxic brain damage. To protect itself from such damage, the brain rapidly uptakes extracellular glutamate and aspartic acids from nerves and glial cells [37,38]. Quinolinic acid acts selectively on the methyl-d,l-aspartate type of amino acid receptor and consequently produces excitation of the central neurons [23,39]. Quinolinic acid requires putative glutamate pathways to produce neurotoxic effects [40]. Connick et al. demonstrated the release of aspartate and glutamate by quinolinate in vivo. This mechanism may explain the induction of neurotoxic effects accelerated by quinolinate [30]. Therefore, elevated levels of aspartic acid in the treated groups may indicate neuroprotection of the organism as a response to 5F-APINAC administration.

Additionally, several metabolites from the different tryptophan catabolism pathways were affected after 5F-APINAC administration. Interestingly, we did not observe any significant differences in the levels of serotonin, the major neuromodulator usually affected by cannabinoids [19]. Blood serotonin is produced by specific intestine cells and released into the circulation, but, generally, the levels of serotonin in the blood are not directly associated with the production of serotonin in the brain. However, both brain and blood serotonin have been reported to be similarly regulated by endogenous processes, including key enzymes, as well as the uptake, transport and storage mechanisms of serotonin [41]. Besides the aforementioned main metabolic pathways of tryptophan, it has been recently shown that tryptophan metabolism occurs also in the gut, being supported by bacterial tryptophanases. Tryptophan is also identified as a key intermediate of host gut microbiome signaling, and its bioavailability is associated with the microbial balance within the gut [42]. Thus, gut microbiota-derived tryptophan metabolites are suggested to mediate gut–brain communications, acting as major mediators possessing neuroprotective properties [43]. In the present study, elevated levels of indole-3-propionic acid and indole-3-carboxaldehyde were found in the rabbits treated with 5F-APINAC. It was suggested that indole-3-propionic acid, acting as a free-radical scavenger, may reduce the oxidative stress in Alzheimer’s disease associated with β-amyloid formation, as well as the neuronal damage [44,45]. On the contrary, the levels of indole-3-lactic acid were found to decrease with a pattern similar to tryptophan, characterized by an extreme fall in the first two hours and a subsequent increase of the metabolite concentrations to reach the basal levels as a U shape.

Due to ethical reasons, there are several restrictions concerning controlled SC administration experiments in humans. Thus, controlled human studies for pharmacokinetic and toxicity evaluations of SCs are not conducted, and only several studies performed in experimental animals have been published. In terms of extrapolation, the pharmacokinetic properties of 5F-APINAC characterized after intravenous administration in the rabbit model may serve as a tool for the prediction of 5F-APINAC pharmacokinetics near to what we expect would happen in humans. We found important changes in plasma concentrations of metabolites related to the kynurenine pathway, i.e., kynurenine, kynurenic acid and quinolinic acid, which probably are at the level of being neurotoxic. Overall, the kynurenine pathway takes an essential role in several fundamental physiological processes, being a mediator of the interactions between the immunological and neuronal functions of the body that are part of the pathophysiology of many diseases. Future experiments considering behavioral and neurotoxic functional outcomes and, also, other biological tissues, such as the brain and cerebrospinal fluid, are warranted to be performed to confirm the degree of neurotoxicity.

## 4. Materials and Methods

### 4.1. Study Design

With a perspective of reducing to a minimum the use of animals in experiments not directly addressed by the development of therapeutic drugs, the present experiment was performed with twelve male rabbits with body weights ranging from 2.5 kg to 3.5 kg after two weeks of quarantine. Animals were housed in standard cages in a temperature-controlled room and did not receive food for 24 h before the administration of the compound and during the experiment. Rabbits were randomly and equally divided into four groups. The first three groups received 1-mL 5F-APINAC intravenously (diluted in DMSO:PEG400:water/5:45:50) at concentration doses of 0.1, 1 and 2 mg/kg, respectively. The three intervention groups were compared with a vehicle control group. Blood samples were collected through ear vein catheters right before the injection; at 20 min after administration and 1, 2, 3, 4, 5, 6 and 24 h after administration. Samples were immediately centrifuged to collect plasma and were stored at −80 °C until laboratory analyses at the Laboratory of Pharmacokinetics and Metabolomic Analysis, Sechenov University (Moscow, Russia). All the experiments and care of the animals were carried out in compliance with the Guidelines for the Use of Laboratory Animals (ISO 33216–2014) “European Convention for the Protection of Vertebrate Animals used for experiments or other scientific purposes” CETS No. 123.

### 4.2. Chemicals and Reagents

Acetonitrile was purchased from J.T. Backer (Deventer, the Netherlands) and ultra-pure water from Biosolve (CE Valkenswaard, the Netherlands); methanol, bovine serum albumin, sodium chloride, formic acid, 6-hydroxynicotinic acid and ascorbic acid were all obtained from Sigma-Aldrich (St. Louis, MO, USA). Tryptophan metabolite standards, with purity ≥ 98%, were received from Toronto Research Chemicals (North York, ON, Canada). 5F-APINAC, with purity ≥ 98%, was obtained from Cayman Chemical (Ann Arbor, MI, USA).

### 4.3. Pharmacokinetic Assessment

#### 4.3.1. Sample Preparation

The plasma samples were prepared using a rapid protein precipitation method. Briefly, 450 µL of acetonitrile were added to 50 µL of plasma, vortexed and centrifuged for 10 min at 3500 rpm. Fifty microliters of the supernatant were then transferred into an LC-MS vial for the subsequent instrumental analysis.

#### 4.3.2. Instrumental Analysis

The LC-MS analysis was performed using a UPLC ACQUITY system connected to a Xevo TQ-S micro IVD mass spectrometer (Waters Inc., Milford, MA, USA). The separation was performed on a chromatographic column Acclaim RSLC 120 AC18 (2.2-μm particle size, 100-mm length × 2.1-mm i.d.) equipped with a guard pre-column Acquity UPLC^®^ BEH C18 (5-mm length × 2.1-mm i.d.) (Waters Inc., Milford, MA, USA). A gradient elution was carried out, with mobile phases consisting of water containing 0.1% formic acid (mobile phase A) and 100% acetonitrile (mobile phase B). The elution gradient program was set as follows: 0 min, 1% B; 1 min, 1% B; 8 min, 99% B; 9 min, 99% B; 9.1 min, 1% B and 11 min, 1% B. The flow rate was set at 0.5 mL/min, and the temperature was maintained at 40 °C, resulting in a total run time of 11 min. Ions were detected in the selected reaction monitoring (SRM) mode (*m*/*z* 385→*m*/*z* 135).

The pharmacokinetic study included assessment of the selectivity, linearity, lower limit of quantification (LLOQ), inter- and intraday precision and recovery. The selectivity assessment was performed using drug-free rabbit blood samples. Linearity was evaluated by the analysis of six calibration levels ranging from 0.1 ng/mL to 1000 ng/mL. Quality control samples at low-, medium- and high-concentration levels were prepared to assess the intra- and inter-day precision and recovery using calculated mean concentrations and subsequent variation coefficients (CV). CVs and a bias below 15% were considered acceptable. More information concerning the method validation parameters is presented in Supplementary Table S1.

### 4.4. Targeted Metabolomics Profile

#### 4.4.1. Sample Preparation

The sample preparation was performed as follows: 100 µL of plasma (calibrators or QCs) were transferred into 1.5-mL Eppendorf microtubes and spiked with the internal standard (10-µL stock solution of 10-µg/mL 2-hydroxynicotinic acid) and 400 µL of acetonitrile. The mixture was then vortexed and centrifuged for 10 min at 13,000 rpm. Then, 400 µL of the supernatant were transferred into a new Eppendorf and evaporated to dryness in a vacuum centrifuge evaporator Speed Vac (Thermo Fisher, Waltham, MO, USA) at 37 °C. The residues were reconstituted with 100 µL of a solution of 0.02% ascorbic acid in 10% methanol, centrifuged and transferred into the LC-MS vial. Five microliters of the extract were injected into the liquid chromatograph.

#### 4.4.2. Instrumental Analysis

A 1200 liquid chromatograph coupled to a model 6490C mass spectrometer (Agilent Technologies, Palo Alto, CA, USA) was used. Separation was achieved on a Discovery PFP HS F5 column (150-mm length × 2.1-mm i.d., particle size 3 µm) (Supelco Inc, Bellefonte, PA, USA). Mobile phase A consisted of 0.1% formic acid in water, while mobile phase B consisted of 100% acetonitrile. The gradient program was set as follows: 0 min, 1% B; 4 min, 10% B; 9 min, 90% B; 10 min, 90% B; 10.1, 1% B; 12 min, 1% B. The flow rate was set at 0.4 mL/min. The column temperature was set at 40 °C. The electrospray ionization was performed in the positive mode. The MS parameters were: gas temperature, 300 °C; gas flow, 8 L/min; nebulizer gas, 20 psi; sheath gas heater, 300; sheath gas flow, 10 L/min and capillary voltage, 3500 V. All additional SRM transitions are presented in Supplementary Table S2. Quality control and calibration standard samples were used. Validation was carried out in a linear range, with R-squared for all analytes above 0.98. The concentrations of the calibrators were within 15% of their nominal values. The lower limit of quantification (LLOQ) was calculated as the lowest standard on the calibration curve that could quantitatively be determined with high precision. Precision of the method was counted at three concentration levels in six replicates of quality control samples on the same day and three consecutive days, respectively. More information concerning the validation parameters for quantification of the metabolites associated with neurotransmission is presented in Supplementary Table S3.

#### 4.4.3. Statistical Analysis

The pharmacokinetics properties of 5F-APINAC were analyzed by a noncompartmental model using the freely available PK-solver software. The areas under the plasma concentration–time curve (AUCs) were calculated using the trapezoidal rule extrapolated to infinity. The terminal elimination half-life (t1/2) and mean residence time (MRT) were obtained. All data were expressed as the mean ± standard deviation (SD). Normality of the data was tested using the Shapiro–Wilk test. The AUCs and the concentrations profiles for each metabolite in the intervention groups were compared to the vehicle control group using Student’s *t*-tests or the equivalent nonparametric Mann–Whitney *U* tests, depending on their distribution. *p*-values < 0.05 were considered significant. Statistical analyses were performed using the Python SciPy library (http://www.scipy.org/, accessed on 22 June 2021). In addition, heat map visualizations and clustering analysis were performed as complementary analyses using Metaboanalyst 4.0 (http://www.metaboanalyst.ca/ (accessed on 15 May 2021), Wishart Research Group, McGill University, Montreal, QC, Canada).

## 5. Conclusions

The present study reported the pharmacokinetics properties of the new SCs 5F-APINAC and a novel and original analysis of the changes of the plasma profiles of tryptophan metabolites observed in the plasma of rabbits after intravenous administration of this drug of abuse. We confirmed that 5F-APINAC has rapid biotransformation, with a maximum concentration within the first time points after the drug administration, followed by gradual elimination. Significant alterations of the plasma concentrations in metabolites related to the kynurenine metabolic pathway, serotonergic system/serotonin pathway, aspartic acid innervation system and microbial tryptophan catabolism were found. These alterations were indicative of the potential detrimental neurotoxic effects of 5F-APINAC in mammals associated with the toxicity of this drug of abuse. The utilization of more complex biological models and tissues in future studies is advised to expand the present findings on the mechanisms of action and the negative effects of 5F-APINAC in humans.

## Figures and Tables

**Table 1:** Pharmacokinetic properties of 5F-APINAC.

Dosage (mg/kg)	t1/2 (min) ^1^	AUC 0–24 (ng/mL × h) ^1^	MRT 0–24 (min) ^1^	Clearance (mL/(kg × h) ^1^
0.1	74.8 ± 27.5	1737.5 ± 421.9	106.6 ± 39.8	14.2 ± 8
1	47.9 ± 2.1	2957.04 ± 270.4	53.5 ± 1.8	153 ± 2
2	26.0 ± 5.9	4132.5 ± 445.2	24.03 ± 6.9	324 ± 5

^1^ Data represent the mean ± standard deviation (SD).

**Table 2:** Summary of the main findings for the targeted metabolites associated with neurotransmission.

Neurotransmitter System	Metabolite	Role	Trend		
			Low Dose ^1^	Medium Dose ^1^	High Dose ^1^
Gamma-aminobutyric acid/glutamic acid innervation	Gamma-aminobutyric acid	Neurotransmitter	No trend		
	Glutamic acid	Neurotransmitter, precursor	No trend		
	Glutamine	Precursor	No trend		
Serotonergic system/serotonin pathway	Tryptophan	Precursor	↓	↓	--
	Serotonin	Neurotransmitter	No trend		
	5-Hydroxytryptophan	Precursor	No trend		
	5-Hydroxyl indole acetic acid (5-HIAA)	Metabolite	↓	--	↓
	Tryptamine	Metabolite	No trend		
Dopaminergic/ adrenergic system	Phenylalanine	Precursor	No trend		
	Tyrosine	Precursor	No trend		
	L-DOPA	Precursor	No trend		
	Norepinephrine	Neurotransmitter	No trend		
	Epinephrine	Neurotransmitter	No trend		
	Metanephrine	Metabolite	No trend		
Aspartic acid innervation system	Aspartic acid	Neurotransmitter	↑	↑	↑
	Asparagine	Precursor	No trend		
Cholinergic system	Acetylcholine	Neurotransmitter	No trend		
	Choline	Precursor	No trend		
Kynurenine pathway	Kynurenine	Tryptophan conversion	↑	--	--
	Kynurenic acid	Tryptophan conversion	--	↑	↑
	Xanthurenic acid	Tryptophan conversion	↑	↑	↑
	Quinolinic acid	Tryptophan conversion	↑	↑	↑
	Anthranilic acid	Tryptophan conversion	↓	↓	↓
	Picolinic acid	Tryptophan conversion	No trend		
Microbial tryptophan catabolism	Indole-3-propionic acid	Tryptophan conversion	--	--	↑
	Indole-3-carboxaldehyde	Tryptophan conversion	↑	↑	↑
	Indole-3-acetic acid	Tryptophan conversion	No trend		
	Indole-3-butyric acid	Tryptophan conversion	No trend		
	Indole-3-lactic acid	Tryptophan conversion	↓	↓	↓
	Indole-3-acrylic acid	Tryptophan conversion	No trend		

^1^ ↑—increased; ↓—decreased.

## Data Availability

Data is contained within the article and Supplementary Material.

## Supplementary Materials

The following are available online at https://www.mdpi.com/article/10.3390/ph14070668/s1, Table S1: Validation parameters of the pharmacokinetic profiling of 5F-APINAC, Table S2: MRM transitions of endogenous metabolites, Table S3: Validation parameters for the quantitative analysis of endogenous metabolites, Figure S1: Heat map visualization and cluster analysis of the significantly changed endogenous tryptophan metabolites over time for three different injected doses. Each cell provides increased (red) or decreased (blue) mapping of the tested animals.

## References

[1] Savchuk S., Appolonova S., Pechnikov A., Rizvanova L., Shestakova K., Tagliaro F. (2017). In Vivo metabolism of the new synthetic cannabinoid APINAC in rats by GC–MS and LC–QTOF-MS. Forensic Toxicol..

[2] Zangani C., Schifano F., Napoletano F., Arillotta D., Gilgar L., Guirguis A., Vento A. (2020). The e-psychonauts’ ‘Spiced’ World; assessment of the synthetic cannabinoids’ information available online. Curr. Neuropharmacol..

[3] Alves V.L., Gonçalves J.L., Aguiar J., Teixeira H.M., Câmara J.S. (2020). The synthetic cannabinoids phenomenon: From structure to toxicological properties. A review. Crit. Rev. Toxicol..

[4] Alipour A., Patel P.B., Shabbir Z., Gabrielson S. (2019). Review of the many faces of synthetic cannabinoid toxicities. Ment. Health Clin..

[5] Szabo B., Schlicker E. (2005). Effects of Cannabinoids on Neurotransmission.

[6] Watkins A.R. (2019). Cannabinoid interactions with ion channels and receptors. Channels.

[7] Appolonova S., Palacio C., Shestakova K., Mesonzhnik N., Brito A., Kuznetsov R.M., Markin P.A., Bochkareva N.L., Burmykin D., Ovcharov M. (2019). In Vivo and invitro metabolism of the novel synthetic cannabinoid 5F-APINAC. Forensic Toxicol..

[8] Markin P.A., Brito A., Moskaleva N.E., Tagliaro F., La Frano M.R., Savitskii M.V., Appolonova S.A. (2021). Short- and long-term exposures of the synthetic cannabinoid 5F-APINAC induce metabolomic alterations associated with neurotransmitter systems and embryotoxicity confirmed by teratogenicity in zebrafish. Comp. Biochem. Physiol. Part C Toxicol. Pharmacol..

[9] Vikingsson S., Gréen H., Brinkhagen L., Mukhtar S., Josefsson M. (2016). Identification of AB-FUBINACA metabolites in authentic urine samples suitable as urinary markers of drug intake using liquid chromatography quadrupole tandem time of flight mass spectrometry. Drug Test. Anal..

[10] Thomsen R., Nielsen L.M., Holm N.B., Rasmussen H.B., Linnet K., INDICES Consortium (2015). Synthetic cannabimimetic agents metabolized by carboxylesterases. Drug Test. Anal..

[11] Andersson M., Diao X., Wohlfarth A., Scheidweiler K.B., Huestis M.A. (2016). Metabolic profiling of new synthetic cannabinoids AMB and 5F-AMB by human hepatocyte and liver microsome incubations and high-resolution mass spectrometry. Rapid Commun. Mass Spectrom..

[12] Moskaleva N.E., Baranov P.A., Mesonzhnik N.V., Appolonova S.A. (2017). HPLC–MS/MS method for the simultaneous quantification of desmethylmebeverine acid, mebeverine acid and mebeverine alcohol in human plasma along with its application to a pharmacokinetics study. J. Pharm. Biomed. Anal..

[13] Appolonova S.A., Dikunets M.A., Rodchenkov G.M. (2008). Possible indirect detection of rHuEPO administration in human urine by high-performance liquid chromatography tandem mass spectrometry. Eur. J. Mass Spectrom..

[14] Lovelace M.D., Varney B., Sundaram G., Lennon M.J., Lim C.K., Jacobs K., Guillemin G.J., Brew B.J. (2017). Recent evidence for an expanded role of the kynurenine pathway of tryptophan metabolism in neurological diseases. Neuropharmacology.

[15] Wang T., Sun X., Qin W., Zhang X., Wu L., Li Y., Zhou C., Zhou H., He S., Cong H. (2019). From inflammatory reactions to neurotransmitter changes: Implications for understanding the neurobehavioral changes in mice chronically infected with Toxoplasma gondii. Behav. Brain Res..

[16] Vondroušová J., Mikoška M., Syslová K., Böhmová A., Tejkalová H., Vacek L., Kodym P., Krsek D., Horáček J. (2019). Monitoring of kynurenine pathway metabolites, neurotransmitters and their metabolites in blood plasma and brain tissue of individuals with latent toxoplasmosis. J. Pharm. Biomed. Anal..

[17] Jenny M., Santer E., Pirich E., Schennach H., Fuchs D. (2009). Delta9-tetrahydrocannabinol and cannabidiol modulate mitogen-induced tryptophan degradation and neopterin formation in peripheral blood mononuclear cells in vitro. J. Neuroimmunol..

[18] Eskelund A., Li Y., Budac D.P., Müller H.K., Gulinello M., Sanchez C., Wegener G. (2017). Drugs with antidepressant properties affect tryptophan metabolites differently in rodent models with depression-like behavior. J. Neurochem..

[19] Jenny M., Schröcksnadel S., Überall F., Fuchs D. (2010). The Potential Role of Cannabinoids in Modulating Serotonergic Signaling by Their Influence on Tryptophan Metabolism. Pharmaceuticals.

[20] Hu L.J., Li X.F., Hu J.Q., Ni X.J., Lu H.Y., Wang J.J., Huang X.N., Lin C.X., Shang D.W., Wen Y.G. (2017). A Simple HPLC-MS/MS Method for Determination of Tryptophan, Kynurenine and Kynurenic Acid in Human Serum and its Potential for Monitoring Antidepressant Therapy. J. Anal. Toxicol..

[21] Speciale C., Hares K., Schwarcz R., Brookes N. (1989). High-affinity uptake of L-kynurenine by a Na^+^-independent transporter of neutral amino acids in astrocytes. J. Neurosci. Off. J. Soc. Neurosci..

[22] Fukui S., Schwarcz R., Rapoport S.I., Takada Y., Smith Q.R. (1991). Blood-brain barrier transport of kynurenines: Implications for brain synthesis and metabolism. J. Neurochem..

[23] Stone T.W., Connick J.H. (1985). Quinolinic acid and other kynurenines in the central nervous system. Neuroscience.

[24] Dong-Ruyl L., Sawada M., Nakano K. (1998). Tryptophan and its metabolite, kynurenine, stimulate expression of nerve growth factor in cultured mouse astroglial cells. Neurosci. Lett..

[25] Moroni F. (1999). Tryptophan metabolism and brain function: Focus on kynurenine and other indole metabolites. Eur. J. Pharmacol..

[26] Stone T.W. (2000). Development and therapeutic potential of kynurenic acid and kynurenine derivatives for neuroprotection. Trends Pharmacol. Sci..

[27] Hilmas C., Pereira E.F., Alkondon M., Rassoulpour A., Schwarcz R., Albuquerque E.X. (2001). The brain metabolite kynurenic acid inhibits alpha7 nicotinic receptor activity and increases non-alpha7 nicotinic receptor expression: Physiopathological implications. J. Neurosci. Off. J. Soc. Neurosci..

[28] Young H.S., Herbette L.G., Skita V. (2003). Alpha-bungarotoxin binding to acetylcholine receptor membranes studied by low angle X-ray diffraction. Biophys. J..

[29] Ramos-Chávez L.A., Lugo Huitrón R., González Esquivel D., Pineda B., Ríos C., Silva-Adaya D., Sánchez- Chapul L., Roldán-Roldán G., Pérez de la Cruz V. (2018). Relevance of Alternative Routes of Kynurenic Acid Production in the Brain. Oxidative Med. Cell. Longev..

[30] Connick J.H., Stone T.W. (1988). Quinolinic acid effects on amino acid release from the rat cerebral cortex In Vitro and In Vivo. Br. J. Pharmacol..

[31] Stone T.W., Perkins M.N. (1981). Quinolinic acid: A potent endogenous excitant at amino acid receptors in CNS. Eur. J. Pharmacol..

[32] Muller F.L., Song W., Jang Y.C., Liu Y., Sabia M., Richardson A., Van Remmen H. (2007). Denervation-induced skeletal muscle atrophy is associated with increased mitochondrial ROS production. Am. J. Physiology. Regul. Integr. Comp. Physiol..

[33] Pierozan P., Zamoner A., Soska A.K., Silvestrin R.B., Loureiro S.O., Heimfarth L., e Souza T.M., Wajner M., Pessoa-Pureur R. (2010). Acute intrastriatal administration of quinolinic acid provokes hyperphosphorylation of cytoskeletal intermediate filament proteins in astrocytes and neurons of rats. Exp. Neurol..

[34] Sundaram G., Brew B.J., Jones S.P., Adams S., Lim C.K., Guillemin G.J. (2014). Quinolinic acid toxicity on oligodendroglial cells: Relevance for multiple sclerosis and therapeutic strategies. J. Neuroinflammation.

[35] Kerr S.J., Armati P.J., Guillemin G.J., Brew B.J. (1998). Chronic exposure of human neurons to quinolinic acid results in neuronal changes consistent with AIDS dementia complex. AIDS.

[36] Chen Y., Brew B.J., Guillemin G.J. (2011). Characterization of the kynurenine pathway in NSC-34 cell line: Implications for amyotrophic lateral sclerosis. J. Neurochem..

[37] Drejer J., Larsson O.M., Schousboe A. (1982). Characterization of L-glutamate uptake into and release from astrocytes and neurons cultured from different brain regions. Exp. Brain Res..

[38] Sanni L.A., Thomas S.R., Tattam B.N., Moore D.E., Chaudhri G., Stocker R., Hunt N.H. (1998). Dramatic changes in oxidative tryptophan metabolism along the kynurenine pathway in experimental cerebral and noncerebral malaria. Am. J. Pathol..

[39] Stone D.M., Merchant K.M., Hanson G.R., Gibb J.W. (1987). Immediate and long-term effects of 3,4-methylenedioxymethamphetamine on serotonin pathways in brain of rat. Neuropharmacology.

[40] Schwarcz R., Foster A.C., French E.D., Whetsell W.O., Köhler C. (1984). Excitotoxic models for neurodegenerative disorders. Life Sci..

[41] Masters R.D., McGuire M.T. (1994). The Neurotransmitter Revolution: Serotonin, Social Behavior, and the Law.

[42] Agus A., Planchais J., Sokol H. (2018). Gut Microbiota Regulation of Tryptophan Metabolism in Health and Disease. Cell Host Microbe.

[43] Wong C.B., Tanaka A., Kuhara T., Xiao J.Z. (2020). Potential Effects of Indole-3-Lactic Acid, a Metabolite of Human Bifidobacteria, on NGF-induced Neurite Outgrowth in PC12 Cells. Microorganisms.

[44] Karbownik M., Reiter R.J., Garcia J.J., Cabrera J., Burkhardt S., Osuna C., Lewiński A. (2001). Indole-3-propionic acid, a melatonin-related molecule, protects hepatic microsomal membranes from iron-induced oxidative damage: Relevance to cancer reduction. J. Cell Biochem..

[45] Chyan Y.J., Poeggeler B., Omar R.A., Chain D.G., Frangione B., Ghiso J., Pappolla M.A. (1999). Potent neuroprotective properties against the Alzheimer beta-amyloid by an endogenous melatonin-related indole structure, indole-3-propionic acid. J. Biol. Chem..

