# Supplementary material for: Pharmacokinetic Properties of the Novel Synthetic Cannabinoid 5F-APINAC and Its Influence on Metabolites Associated with Neurotransmission in Rabbit Plasma
Source: Pharmaceuticals (Basel). 2021 Jul 13;14(7):668. doi: 10.3390/ph14070668 (PMC8308683; doi:10.3390/ph14070668)

Supplementary Table S1. Validation parameters of the pharmacokinetic profiling of 5F-APINAC

Target compound	Linearity (ng/ml)	R^2	LLOQ (ng/ml)	Intra-day precision	Inter-day precision	% Recovery, ±RSD
5F-APINAC	0.1-1000	0.9997	0.1	8.6	10.6	98

LLOQ – Low limit of quantification
RSD – Relative Standard Deviation

Supplementary Table S2. MRM transitions of endogenous metabolites.

Target compound	RT (min)	Precursor ion	Product ion	Fragmentor	Collision energy (eV)
2-Aminobutyric acid	1.2	104	58	90	10
3-Aminoisobutyric acid	1.5	104	57	90	15
4-Aminobutyric acid	1.4	104	87	90	10
Glutamic acid	1.1	148	84	100	15
			56	100	30
Glutamine	1.0	147	130	100	10
			84	100	15
Tryptophan	6.4	205.1	146.1	100	20
Serotonin 1	5.6	177.1	160.1	90	10
Serotonin 2	5.6	160.1	132.1	120	20
			105.1	120	30
5-Hydroxytryptophan	4.8	221	160	90	20
			162	90	20
HIAA	6.1	192.1	146.1	100	20
Tryptamine	8.0	161,1	144,1	95	10
			115.1	95	30
Tyrosine	3.1	182	165	90	5
			136	90	10
L-DOPA	2.3	198	181	100	10
			152	100	10
Epinephrine	2.3	184	166	90	5
			151	90	25
Norepinephrine	1.6	170	152	90	5
Metanephrine	4.5	198	180	90	5
Normetanephrine	3.0	166	134	100	15
Aspartic acid	1.0	134	88	100	10
			76	100	15
Asparagine	1.0	133	87	100	10
			84	100	20
Acetylcholine	4.5	146	87	90	15
			43	90	10
Kynurenine	4,6	209,1	146	95	30
			94	95	10
Kynurenic acid	6,6	190,1	144,1	95	20
Xanturenic acid	6.6	206	160	95	20
			160	95	30
Quinolinic acid	1.8	168,1	106.1	95	15
			78.1	95	30
Antranillic acid	6,5	138,1	120,1	95	10
			92	95	20
Indol-3-butyric acid	7.76	204.2	144.2	100	25
			130.1	100	25
Indole-3-propionic acid	7.5	190,1	130,1	95	20
Indol-3-acrilyc acid	7.4	188.1	115.1	100	25
Indol-3-acetic acid	7.2	176,1	130,1	95	20
Indole-3-carboxaldehyde	7.3	146,1	118,1	95	15
			91,1	95	30
DL-Indole-3-lactic Acid	6.8	206,1	118,1	95	22

Citrulline	1.1	176.1	113.1	90	20
			70.1	90	30
Choline	1.6	104	60	90	5

Supplementary Table S3. Validation parameters for quantitative analysis of endogenous metabolites.

**Target compound**	**Linearity (ng/ml)**	**R^2**	**LLOQ (ng/ml)**	**Intra-day precision**	**Inter-day precision**	**% Recovery, ±RSD**
Asparagine	1-1000	0.997	1	6.4	10.1	91
2-Aminobutyric acid	1-1000	0.998	1	7.3	9.5	94
3-Aminoisobutyric acid	1-1000	0.997	1	10.6	11.2	89
4-Aminobutyric acid	1-500	0.998	1	10.1	12.3	93
5-Hydroxytryptophan	1-1000	0.998	1	9.6	14.5	96
Acetylcholine	0.1-100	0.994	0.1	5.1	8.5	97
Antranillic acid	1-1000	0.994	1	7.3	9.3	95
Aspartic acid	10-1000	0.996	10	8.3	10.1	93
Biopterin	1-500	0.997	1	9.5	11.8	94
Choline	1000-100000	0.998	1000	10.2	13.4	90
Citrulline	50-5000	0.995	50	9.3	14.6	98
Cortisol	10-10000	0.998	10	11.2	13.5	99
Epinephrine	1-500	0.999	1	10.5	12.8	99
Glutamic acid	10-5000	0.998	10	9.3	11.9	94
Glutamine	100-5000	0.998	100	7.9	10.4	92
HIAA	1-10000	0.999	1	5.7	9.2	95
Indole-3-acetic acid	10-1000	0.999	10	5.4	9.8	93
Indole-3-acrylic acid	1-1000	0.999	1	6.2	11.4	92
Indole-3-butyric acid	0.1-100	0.999	0.1	8.4	10.9	95
Indole-3-carboxaldehyde	1-1000	0.998	1	6.9	7.4	93
Indole-3-lactic acid	10-10000	0.998	10	5.7	8.8	96
Indole-3-propionic acid	1-1000	0.999	1	7.4	9.5	91
Indole	1-1000	0.999	1	6.1	12.4	98
Kynurenic acid	10-10000	0.999	10	8.5	11.1	93
Kynurenine	10-50000	0.999	10	7.5	10.8	95
L-DOPA	1-1000	0.999	1	9.2	14.2	97
Metanephrine	10-5000	0.998	10	8.7	12.9	94
Norepinephrine	1-1000	0.998	1	10.3	13.1	92
Normetanephrine	1-500	0.996	1	9.2	10.8	95
Quinolinic acid	1-1000	0.997	1	7.3	9.5	94
Serotonine 1	10-50000	0.997	10	8.4	10.6	92
Serotonine 2	10-25000	0.998	10	6.3	9.3	91
Tryptamine	0.1-100	0.998	0.1	5.2	9.7	96
Tryptophan	10-1000 μg/ml	0.999	10 μg/ml	9.7	11.4	94
Tyrosine	1-100 μg/ml	0.997	1 μg/ml	9.3	12.9	95
Xanturenic acid	1-1000	0.999	1	12.0	13.7	98
Neopterine	0.1-100	0.999	0.1	9.4	11.2	93
Picolinic acid	1-1000	0.997	1	8.3	10.8	99
Phenylalanine	1-100 μg/ml	0.996	1000	7.5	9.9	92

LLOQ – Low limit of quantification
RSD – Relative Standard Deviation

Supplementary Figure S1. Heat map visualization and cluster analysis of the significantly changed endogenous tryptophan metabolites over time for three different injected doses. Each cell provides increased (red) or decreased (blue) mapping of the tested animals. a. Comparison between vehicle control rabbits and rabbits administered with 0.1 mg/kg of 5F-APINAC; b. Comparison between vehicle control rabbits and rabbits administered with 1 mg/kg of 5F-APINAC; c. Comparison between vehicle control rabbits and rabbits administered with 0.1 mg/kg of 5F-APINAC; d. Cluster analysis of the discriminated metabolites.

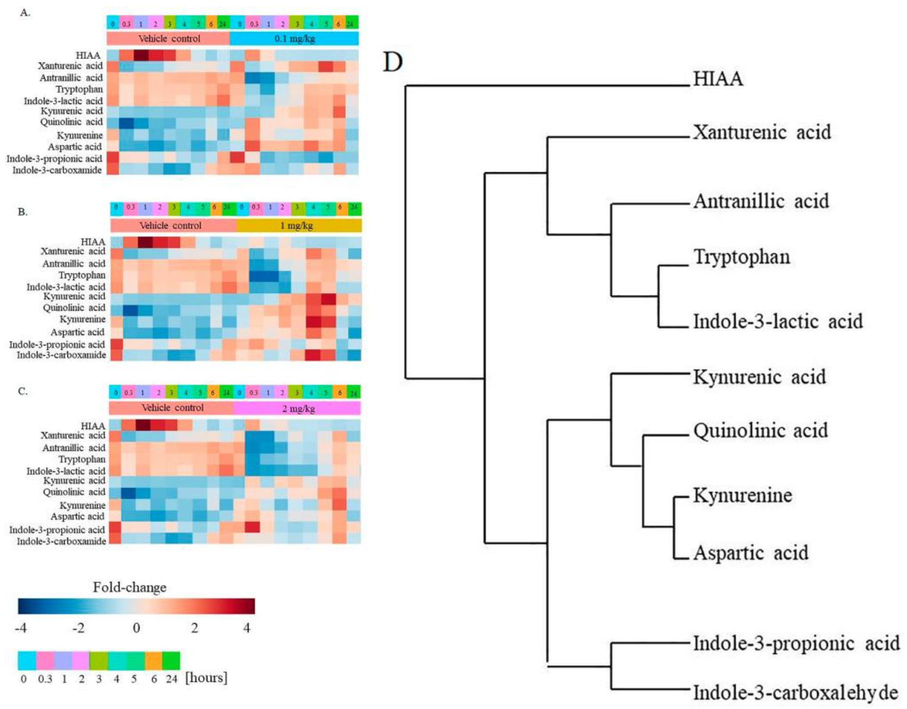

Supplement: Supplementary file 1 [file pharmaceuticals-14-00668-s001.zip › pharmaceuticals-1274244-supplementary.pdf]
